# Microvolume Protein Concentration Determination using the NanoDrop 2000c Spectrophotometer

**DOI:** 10.3791/1610

**Published:** 2009-11-04

**Authors:** Philippe Desjardins, Joel B. Hansen, Michael Allen

**Affiliations:** Thermo Scientific NanoDrop Products

## Abstract

Traditional spectrophotometry requires placing samples into cuvettes or capillaries. This is often impractical due to the limited sample volumes often used for protein analysis. The Thermo Scientific NanoDrop 2000c Spectrophotometer solves this issue with an innovative sample retention system that holds microvolume samples between two measurement surfaces using  the surface tension properties of liquids, enabling the quantification of samples in volumes as low as 0.5-2 μL. The elimination of cuvettes or capillaries allows real time changes in path length, which reduces the measurement time while greatly increasing the dynamic range of protein  concentrations that can be measured. The need for dilutions is also eliminated, and preparations for sample quantification are relatively easy as the measurement surfaces can be simply wiped with laboratory wipe. This video article presents modifications to traditional protein concentration determination methods for quantification of microvolume amounts of protein using A280 absorbance readings or the BCA colorimetric assay.

**Figure:**
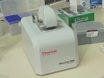


## Protocol

### I. The NanoDrop 2000c Spectrophotometer

NanoDrop technology is based on an innovative sample retention system that uses the surface tension to hold and measure microvolume samples between two optical pedestals without the use of cuvettes or capillaries. The NanoDrop 2000c spectrophotometer uses this technology to quickly and easily measure 0.5-2 μL droplets of proteins, DNA, RNA, and other biomolecules. This capability has become increasingly important as molecular techniques continually evolve to use smaller amounts of material for analysis. The microvolume spectrophotometer ideal for conditions in which sample is limited. However, the ease-of-use, fast measurement cycle and, and extensive concentration range also make the spectrophotometer suitable when ample amounts of sample are available. The measurement cycle is also greatly reduced, helping scientists increase efficiency throughout their workflows.

The microsample is placed directly on top of the detection surface and a liquid column is created between the ends of the optical fibers by surface tension. This liquid column forms a vertical optical path. A xenon flash lamp provides the light source and a spectrometer utilizing a linear CCD array is used to analyze the light that passes through the sample.

Removing traditional containment devices such as cuvettes from the system has several advantages: very small amounts of sample are needed for measurement, cleanup simply requires wiping the optical surfaces with a laboratory wipe, and the path length can be changed in real time during the measurement.

The NanoDrop 2000c determines the optimal path length automatically (1 mm to 0.05 mm), providing the most extensive range of possible protein concentration measurements without dilutions. By shortening the path length, higher concentrations of protein can be measured. This effectively removes the need to perform dilutions for most protein samples. For example, the NanoDrop 2000c can measure BSA concentrations as high as 400 mg/mL.

The NanoDrop 2000c Spectrophotometer is a full spectrum spectrophotometer for measuring the absorbance of DNA, RNA, proteins, and other biomolecules. This video protocol will focus on the measurement of proteins.

### II. Microvolume Protein Concentration Determination Using A280 Absorbance Measurements

#### a. Principle of A280 Measurements

The Protein A280 method is applicable to purified proteins that contain Tryptophan, Tyrosine, Phenylalanine residues or Cysteine-Cysteine disulphide bonds and exhibit absorbance at 280 nm. This method uses the A280 absorbance value in combination with either the mass extinction coefficient or the molar extinction coefficient to calculate the concentration of the purified protein. The advantage of direct A280 measurements is that the generation of a standard curve is not required to determine protein concentration. If the sample is an uncharacterized protein solution, cell lysate, or crude protein extract, then using one of the pre-configured colorimetric methods available on the NanoDrop 2000/2000c, such as BCA, Pierce 660 nm, Bradford, and Lowry assays, is recommended.

#### b. Microvolume Protein A280 Measurements - Startup

1. Select the Protein A280 application from the main menu.
2. Select the type of sample to be measured from the drop-down list. The default setting is recommended for most unknown protein mixtures in which 1 Abs = 1 mg/mL. If measuring a previously characterized purified protein, then either the mass extinction coefficient or molar extinction coefficient and molecular weight may be entered to determine the protein concentration more precisely.
3. Choose the concentration units from the drop-down list.

#### c. Microvolume Protein A280 Measurements - Blanking

1. Establish a blank using an appropriate buffer. It is important to use the buffer in which the protein is suspended. The buffer used should be the same pH and of a similar ionic strength as the sample solution. Note: RIPA buffers contribute too much absorbance at 280 nm and are therefore incompatible with direct A280 protein measurements. For proteins suspended in a RIPA buffer, it is recommended protein concentration be determined using a RIPA buffer compatible colorimetric assay.
2. Pipette 2 μL of the appropriate blanking solution onto the bottom pedestal, lower the arm and click Blank.
3. Wipe the blank solution from the lower and upper pedestals using a dry laboratory wipe.

#### d. Microvolume Protein A280 Measurements   Measuring

Important considerations: The homogeneity of the sample is extremely important since such a small volume is being measured. To ensure that samples are homogenous, gently but thoroughly mix the samples immediately prior to taking an aliquot for measurement. Avoid introducing bubbles when mixing and pipetting.

Due to the variability in surface tension between different proteins, we recommend loading 2 μL of samples to ensure proper column formation. Always use low retention pipette tips. To load a sample, touch the pipette tip to the lower optical pedestal surface while expelling the solution to prevent the solution from adhering to the outside of the pipette tip. Expel less than the full amount of sample to prevent blowout and introduction of bubbles in the sample.

1. Enter a sample ID in the appropriate field, load 2 μL of the first sample as described for the blank and click Measure. A fresh 2μL aliquot of sample should be used for each measurement.
2. Remove the sample from the lower and upper pedestals using a dry laboratory wipe.

#### e. Microvolume Protein A280 Measurements   Cleaning

An ordinary, lint-free, laboratory wipe is often sufficient for cleaning the optical pedestals between measurements.

#### f. Making Protein A280 Measurements   Reconditioning

Solutions and reagents containing surfactants may uncondition the measurement pedestal surfaces over time, preventing the sample liquid column to form. A flattening of the droplet on the lower pedestal is indicative of the optical surface becoming unconditioned. If the surface properties have been compromised, reconditioning the pedestals is important to ensure sample column formation. If this occurs, buff the optical surfaces vigorously using laboratory wipe or use NanoDrop Pedestal Reconditioning Compound (PR-1) as directed.

### III. Microvolume Protein Concentration Determination Using Colorimetric Assays

#### a. Principle of colorimetric detection

The NanoDrop 2000c spectrophotometer can also be used to measure uncharacterized protein solutions, cell lysates, and crude protein extracts using colorimetric assays.

Colorimetric methods are indirect methods that involve interaction of a dye with the protein component of the sample to produce a new complex that absorbs light in the visible wavelength range.

The NanoDrop 2000c spectrophotometer has several pre-configured colorimetric assays including BCA, Pierce 660, Bradford, and Lowry methods. The BCA assay will be demonstrated as an example colorimetric assay using a microvolume spectrophotometer. (screenshot)

The BCA (Bicinchoninic Acid) assay is a common colorimetric method often used for dilute protein solutions and proteins in the presence of components that have significant UV (280 nm) absorbance. Unlike the Protein A280 method, the Protein BCA method requires that a standard curve be generated before sample protein concentrations can be measured.

The method uses bicinchoninic acid (BCA) as the detection reagent. The Cu-BCA chelate formed in the presence of protein is measured at 562 nm and normalized at 750 nm.

#### b. Microvolume BCA Assay Measurements   BCA Assay Preparation

1. The required reagents are contained in the reducing agent compatible kit (Thermo Scientific Pierce cat. no. 23250): BCA reagent A, BCA reagent B, Compatibility reagent, Reconstitution buffer, and Albumin (BSA) standards.
2. Equilibrate all unknown proteins and protein standards to room temperature and mix thoroughly.
3. Prepare enough fresh working reagent for the standards and samples to be measured using a 50:1 ratio of reagent A:B
4. For the micro assay, use a 1:1 ratio of working  reagent to sample.  Add 10 μL of working reagent to each tube with enough tubes to cover all samples and standards. For the high-range assay, use a 20:1 ratio of working reagent to sample.  Add 190 μL of working reagent to each tube with enough tubes to cover all samples and standards.  Refer to the Pierce BCA kit literature to determine which ratio is suitable for your samples.
5. Add 10 μL of standard or sample to each tube containing reagent. Mix well by gently vortexing.
6. Incubate the tubes at 37 °C for 30 minutes.
7. Allow the reactions to equilibrate to room temperature (~10 minutes).

#### c. Microvolume BCA Assay Measurements   Generating Standard Curve

1. Select the Protein BCA method from the main menu. If the wavelength verification window appears, ensure the arm is down.
2. Enter the values for each standard concentration in the right pane table. The software allows for the reference and up to 7 additional standards. The Reference and/or standards should be measured in replicates. Note: The minimum requirement for standard curve generation is the measurement of two standards or the measurement of the zero reference and at least one standard. It is recommended that additional standards be included as necessary to cover the expected assay concentration range.
3. Establish a blank. The blank for colorimetric assays is generally deionized H_2_O.
4. Pipette 2 μL of dH_2_O onto the bottom pedestal, lower the arm and click Blank. Only one blank is necessary to cover all subsequent measurements of the reference and standards.
5. Establish the reference by pipetting a 2 μL aliquot containing only working reagent and buffer with no protein onto the lower pedestal. Lower the arm and click Measure.
6. Under the Standards tab, highlight the desired standard and pipette 2 μL of the desired standard onto the lower pedestal. Lower the arm and click Measure. Repeat the process for all standards. Be sure to measure all standards prior to measuring samples. To view standard curve click View Standard Curve.

#### d. Microvolume BCA Assay Measurements   2 μL Protein Measurements

1. After all of the Standards have been measured, click on the Samples button. Enter the sample ID. Load 2 μL of sample onto the lower pedestal and click Measure. A fresh 2 μL aliquot of sample should be used for each measurement.
2. Wipe the sample from the lower and upper pedestals using a dry laboratory wipe.

#### e. Microvolume BCA Assay Measurements   Cleaning and Reconditioning

Perform cleaning and reconditioning as described in the direct A280 absorbance measurements.

### Representative Results:

Microvolume protein concentration determination is performed by either a direct A280 measurement or an indirect colorimetric assay.  The A280 measurement example determines protein concentration based on the extinction coefficient of the protein of interest.  The BCA colorimetric assay example determines protein concentration based of a standard curve of known protein concentrations. 

## Discussion

Microvolume quantitation uses the intrinsic surface tension properties of a sample to form a liquid column between two measurement surfaces.  The absence of a containment device,  allows the path length to change in real time, essentially eliminating the need to perform dilutions.  This capability dramatically increases the dynamic range of protein concentrations as well as the speed of measurement. By using minimal amounts of sample, a large number of samples can be analyzed quickly and accurately, allowing scientists to take more measurements and achieve better quality control. In addition, and if necessary, precious samples can be recovered after taking a measurement. When such small volumes are being measured, sample homogeneity is extremely important to avoid sampling errors. Low retention pipette tips should always be used for loading samples and the tips should be changed between sample replicates. The pedestal surfaces must be properly cleaned and conditioned to ensure the most accurate results. Finally, the microvolume spectrophotometer is ideal when sample is limited, however, the ease-of-use, speed, and extensive dynamic range of the spectrometer make it suitable when sample is plentiful.
